# The exploration of the knowledge, attitudes and practice behaviors of advanced care planning and its related predictors among Taiwanese nurses

**DOI:** 10.1186/s12904-019-0483-9

**Published:** 2019-11-11

**Authors:** Chiu-Chu Hsieh, Hsiang-Ping Huang, Tao-Hsin Tung, I-Chien Chen, Randal D. Beaton, Sui-Whi Jane

**Affiliations:** 1 Department of Nursing, Kaohsiung Armed Forces General Hospital, Kaohsiung, Taiwan, Republic of China; 2 Department of Nursing, Chang Gung University of Science and Technology, Tao-Yuan, Taiwan, Republic of China; 3 Department of Medical Research and Education, Cheng Hsin General Hospital, Taipei, Taiwan, Republic of China; 4 Psychosocial & Community Health and Health Services, Schools of Nursing and Public Health, University of Washington, Seattle, USA; 5 Geriatric and Long-term Care Research Center, Graduate Institute of Nursing, Chang Gung University of Science and Technology, 261, Wen-Hua 1st Rd., Gui-Shan Dist, Tao-Yuan City, 33303 Taiwan, Republic of China; 6 Division of Hematology/Oncology, Department of Internal Medicine, Chang Gung Memorial Hospital, Lin-Ko, Taiwan, Republic of China

**Keywords:** Nurse, Advance care planning (ACP), Knowledge, Attitude, Practice behaviors

## Abstract

**Background:**

Despite the documented and well known patient benefits of ACP, the completion of ACP, only a minority of patients, during the advanced or EOL stage of their illnesses, receive such care. The misconceptions about ACP for healthcare providers, such as nurses, might become potential barriers to the effective implication of ACP. Also, from the transcultural perspective, it is evident essential to explore Taiwanese nurses’ attitudes, knowledge, and actions of ACP. The purposes of this study were to explore the implication of ACP or hospice care for nurses caring for non-cancer chronic illness patients at a regional teaching hospital in Taiwan; and, to identify predictors of those nurses’ knowledge, attitudes, and actions toward ACP.

**Methods:**

This cross-sectional study with a purposive sample of 218 nurses was conducted at a teaching hospital in southern Taiwan. Structured questionnaires were employed and data were analyzed with descriptive statistics, t-test, one-way ANOVAs, Pearson’s correlation and multiple regressions.

**Results:**

16.1% of Taiwanese physicians actively initiated ACP issues or conversations with patients or their family members. Nurses’ attitudes toward ACP were fairly positive but their knowledge about ACP was insufficient and actions of ACP were not positively executed. The predictors of ACP-Knowledge (ACP-K) included position title, education hours and lacking of educational training. The predictors of ACP-Attitude (ACP-A) included ACP-K and “fear of patient or family member not accepting”, whereas ACP-A, position title, “patients do not feel necessary” and “not sure physician’s concern” were the predictors of ACP-Act.

**Conclusion:**

Continuous education and training for nurses regarding ACP needs to be improved by taking those predictors found in this current study into account, and more studies on the nurse’s role in ACP also should be further examined.

**Trial registration:**

KAFGH 106–012. Date of registration 1 May 2017.

## Background

Patients with chronic illnesses, despite progress in treating many chronic ailments, often may experience deterioration in their medical condition during the course of their illnesses. Palliative care, an emerging framework for improving the quality of end-of-life (EOL) care, embraces the active involvement of patients and their family members during the advanced and EOL stages of the illness and to manage the physical, psychosocial and spiritual components of their illness processes [1, 2]. According to the National Consensus Project (NCP) [3], the ethical and legal aspects of advanced care planning (ACP) is one of important elements for palliative care. Over the past two decades, the essential implementation of ACP has shifted from providing advance directive (ADs) documents to enabling effective communication between patients and their caregivers regarding the provision of durable power of attorney for health care, preferences or goals of medical care, and treatment directives or living wills (i.e., do not resuscitate, DNR) [4]. Existing studies have shown that early palliative care or ACP improves patients’ perceptions of progress [5], the efficacy of symptom management [6], enhances their quality of life and decreases the utilization of aggressive medical modalities and, importantly, undergirds patients’ rights and supports their quality of dying [5]. Despite the documented and well known patient benefits of ACP, the completion of ACP or related ADs, only a minority of patients, during the advanced or EOL stage of their illnesses, receive such care. For example, hospice care was reportedly provided for only 5–39% of such patients in the United States [7], 11% in the United Kingdom [8] and 2.2% in Taiwan [9]. Further, only 39% of terminal patients in the US received hospice care, of which 35% of patients died in 7–14 days [10, 11] or within 21 days [12]. Thirty-eight percent of patients were admitted to the intensive care unit within 10 days of their death [13]. Similarly, many patients signed their DNR document on the day of their death, and 95% of such DNR documents were signed for by their family members [14]. In Taiwan, a 2011 study found that only 13–17% of terminal cancer patients received hospice care; 17–19% of Taiwanese patients received hospice care 3 days before death [15], and a 2010 investigation found that up to30% of Taiwanese patients continued to receive active emergency treatment at the EOL [16].

Clearly, a cohesive body of scientific evidence suggests there that are barriers to the effective implication of ACP, including medical personnel’s misconceptions (i.e., ACP focuses only on moribund patients [4], the possibility that discussing ACP makes patients or their families uncomfortable or to lose hope [17], and that nurses do not need to participate in ACP [7]). Regarding the latter barrier, nurses are, in fact, the front line of clinical care and are the health provider in the best position to serve as promoters, messengers, and spokespeople facilitating discussions of ACP among healthcare teams, patients and their families [7, 17, 18]. Thus, the knowledge, attitudes, and behaviours of nurses toward ACP is crucial [19] for the successful implementation of ACP. But the accuracy of nurses or advanced practice nurses’ (APN) knowledge on ACP was only 60% [20]-67% [21], respectively. In addition, other factors that hinder or promote nurses’ effective communication with patients regarding ACP need to take into account, such as their age [8, 22], clinical experience [22], personal experience with ACP [7], and/or continuing education regarding ACP [23]. Other factors potentially influencing the implementation of ACP included lack of time to discuss ACP [24], excessive optimism by family members regarding the poor prognosis of the patient/family member [22], and uncertainty of physicians’ concerns or actions of ACP [25].

To date, ACP-related studies primarily focussed on the effects related to the durable power of attorney, patients’/family members’ perceptions/behaviours, or intensive care unit nurses’ knowledge, attitudes, and behaviours toward ACP [5, 26]. Considering the leading causes of death in Taiwan, nurses, often have opportunities to take care of patients with chronic illness other than cancer. Therefore, exploration of Taiwanese nurses’ knowledge and attitudes toward ACP while taking care for patients with chronic illnesses other than cancer are warranted.

The purposes of this study were to explore the implications of ACP or hospice care for nurses caring for patients with non-cancer chronic illnesses at a regional teaching hospital in Taiwan; and, to identify predictors of those nurses’ knowledge, attitudes, and actions toward ACP.

## Methods

### Study design and setting

This descriptive, cross-sectional study with purposively sampling was conducted at a teaching hospital in southern Taiwan, Kaohsiung Armed Forces General Hospital (KAFGH), from December 2017 to February 2018. To be eligible for inclusion in the study, participants must have been registered nurses, had experience caring for non-cancer terminal patients and currently worked in internal medicine, general practice, or intensive care. Nurses who worked in departments with less terminal patients (e.g. orthopaedics, obstetrics & gynaecology, psychiatry, surgery) were excluded from participation.

### Procedures

This study was approved by the Kaohsiung Armed Forces General Hospital (KAFGH) Institutional Review Board (IRB #106–012). Prospective participants received an explanation of the research purpose and procedure and were given an option to participate and informed of their rights. After obtaining written consent, the research associate provided participants with a battery of questionnaires and unsealed envelopes, and participants were asked to drop their sealed envelopes in collection boxes in their units upon completing the questionnaires.

### Instruments

#### Implication of hospice care and ACP (IHAC)

A 6-item measure describing implication of hospice care and ACP used was primarily adapted from a 7-item measure at Zhou’s study [21]. Four items with a Likert scale of 1 never to 5 always were to explore the implication of ACP, such as ‘In my practice, the oncologist(s) initiates the discussion of ACP’. The fifth question asked participants to respond to the following statement: ‘I estimate that the following percentages of patients receive hospice care prior to dying (i.e., within 2 weeks, 1 month of hospice referral)’. The sixth item was a multiple choice question with a list of 8 potential factors influencing nurses’ discussion of ACP (based on the authors’ clinical experience and literature review) such as ‘lacking related training’ or ‘patients do not feel necessary’.

#### Knowledge, attitude, and actions of ACP (KAAC-ACP)

This 31-item measure used in this study to examine nurse participants’ knowledge, attitudes, and actions of ACP and was adapted from a 34-item measure at Zhou’s study [21]. The overall Cronbach α of this measure in Zhou’s KAAC-ACP measure was 0.70–0.84 with test-retest reliability was 0.74. The Cronbach α of KAAC-ACP measure used in this current study was 0.80.

#### ACP-knowledge (ACP-K)

The ACP-K used in the current study consisted of 9 items. Replies were scored 1 correct answer and 0 incorrect answers, higher scores indicating more accurate knowledge about ACP. The discrimination of ACP-K was 1 (fair) and the degree of difficulty was 0.5 (moderate level) in this current study based on the suggestions by Lee [27] and Yu [28]. According to the suggestion by Hsu, Hurng, & Hungrng [29], Kuder-Richardson Formula 20 (KR-20) 0.374 was considered to be acceptable, thus, the KR-20 of 0.42 in this current study reached to an acceptable level.

#### ACP-attitude (ACP-A)

The ACP-A used to measure attitude toward ACP primarily adapted from Zhou’s study [21], consisting of an 8-item ACP-A-b (belief, i.e., ‘ACP discussion is very important for patients with life-threatening illnesses’), a 6-item ACP-A-s (subjective standards, i.e., ‘I believe it is my responsibility to discuss ACP with patients and families’), a 4-item ACP-A-p (perceived control, i.e., ‘I feel confident in my ability to communicate ‘bad news’) with a Likert scale ranging from 1 (very much agree) to 5 (very much disagree). On this latter measure lower scores indicated more positive attitudes. The Cronbach’s α of the ACP-A measure in this study was 0.73.

#### ACP-action (ACP-act)

The subscale of KAAC-ACP used to measure ACP actions was 4-item ACP-Act (i.e., I routinely initiate ACP discussions with advanced cancer patients) with a Likert scale ranging from 1, very much agree to 5, very much disagree. Lower scores on this measure indicated more positive actions of ACP. The Cronbach’s α of the ACP-Act in this study was 0.86.

#### Demographic information and hospice-related education or experience

Nurses’ personal demographic information solicited in this study included their age, ethnicity, education, position, clinical ladder, and years of clinical practice experience. Nurses’ hospice care or ACP-related information included completed hours of hospice/palliative continuing education, experience with taking care of patients with severe illnesses and whether they had experience taking care of family members with severe illnesses.

### Statistical analysis

All statistical analyses were conducted using SPSS 20 software for Mac. Categorical variables were reported as well as frequency distribution and percentage, whereas continuous variables were reported as numerical values and standard deviations. Inferential analyses included Pearson correlation coefficients used to investigate the relationship between participants’ knowledge, attitude, and actions of ACP and associated continuous factors; and simultaneous regressions model used to assess the independent effects of relevant factors on ACP values after controlling for the covariates. Sample size calculations were performed primarily depending on the regression analyses with 90% power, 11 independent variables, 10% variances, and 0.05 alpha, accounting for a total of 225 participants.

## Results

### Sample characteristics

Of the 225 distributed questionnaires, 221 were returned (98.2% response rate) and the total sample consisted of 218 (98.6% completion rate) with a mean age of 33.4 years (SD = 8.0). The majority of participants were N or N2 nurses (63%), university graduates (73%), and the respondent sample’s averaged years of clinical work experience was 10.8 years (their average years of service at the current institution was 5.8 years) (Table 1).

**Table 1:** Demographic characteristics of the sample (*n* = 218)

Characteristics	*n* (%)		Mean ± SD
Age(years)^a^			33.4 ± 8.0
21~30	92(42.2)		
31~40	91(41.7)		
41~60	35(16.1)		
Ethnicity			
Taiwanese	200(91.7)		
Mainlander	8(3.7)		
Hakka	7(3.2)		
Aborigine	3(1.4)		
Education			
Junior college	48(22.0)		
University	159(72.9)		
Master or higher	11(5.1)		
Marital status			
Single	108(49.5)		
Married	102(46.8)		
Divorced	8(3.7)		
Religion			
None	71(32.6)		
Buddhism /Taoism	121(55.5)		
other	26(11.9)		
Department			
ICU	84(38.5)		
Medical Unit	61(28.0)		
Mixed Unit	46(21.1)		
Surgical Unit	27(12.4)		
Position Title			
Registered Nurse	201(92.2)		
Assistant head nurse	7(3.2)		
Head Nurse	10(4.6)		
Clinical ladder ^a^			
N	77(35.3)		
N1	55(25.2)		
N2	60(27.5)		
N3 & N4	26(11.9)		
Years of work experience ^b^			10.8 ± 8.4
3 years below	45(20.6)		
3 years and higher	173(79.4)		
Hours of Palliative continuous education			
0–6 h	175(81.0)		
7–12 h	31(14.2)		
13–21 h	10(4.7)		
Hospice-related certification			
Basic or advanced training	13(6.0)		
None	205(94.0)		
Have ever serious illness			
Yes	23(10.6)		
No	195(89.4)		
Have signed palliative care directs document			
Yes	8(3.7)		
No	210(96.3)		
Have experience of taking family members with end-stage illness			
Yes	81(37.2)		
No	137(62.8)		

Note: a: According to the years of professional seniority, the registered nurses are classified as four clinical ladders, including N(within 1 year of clinical experience), N1(1 year of clinical experience), N2(2 years of clinical experience), N3(3 years of clinical experience), & N4(4 years of clinical experience) in Taiwan; b: Mean ± SD

### Implication of hospice care and ACP (IHAC)

The implication of hospice care and ACP (IHAC) was evaluated with four items with 1 (never) to 5 (always), and the score of 3 (sometimes) was used as a cut-off point for further comparisons. In this study, there was 16.1% of physicians often or always initiating discussion of ACP with patients (*p* = 0.007) or 13.3% of terminal patients would often or always discuss ACP with their physicians (*p* = 0.001). The difference between “sometimes” and “seldom” frequency of terminal patients receiving critical care in the last month of life was not significant (*p* = 0.255), indicating that the frequency was moderate compared to often or seldom responses. In contrast, terminal patients received relatively frequent hospice care in clinical practice (*p* = 0.001) (Table 2). In addition, the most common timing of hospice referral prior to dying was within 2 weeks (32.3%), followed by within 1 month (26.8%), 3 months (20.3%), and 6 months (16.8%).The frequency of potential factors influencing nurses’ discussion of ACP are given in the Table 3.

**Table 2:** The Implications of Hospice and Advanced Care Planning (ACP) (*n* = 218)

Questions	Never *n* (%)	Seldom *n* (%)	Sometimes *n* (%)	Often *n* (%)	Always *n* (%)	M ± SD	*t*	*p* value
1. In clinical practice, physician______ discuss ACP actively	4(1.8)	56(25.7)	123(56.4)	34(15.6)	1(0.5)	2.87 ± 0.70	−2.708	0.007
2. In my estimation, about ____ % of EOL patients have discussed ACP with clinicians in clinical practice	1(0.5)	74(33.9)	114(52.3)	28(12.8)	1 (0.5)	2.79 ± 0.68	−4.583	< 0.001
3. In my estimation, about ____ % of EOL patients have received critical care during the last month of their lives in clinical practice	3(1.4)	75(34.4)	84(38.5)	46(21.1)	10(4.6)	2.93 ± 0.89	−1.141	0.255
4. In my estimation, about ____% of EOL patients received hospice care in clinical practice	1(0.5)	33(15.1)	118(54.1)	63(28.9)	3(1.4)	3.16 ± 0.70	3.284	0.001

Note: EOL, end of life; these four questions were assessed with a Likert scale of 1 never to 5 always

**Table 3:** Potential factors influencing nurses’ discussion of ACP (*n* = 218)

Items	*n*(%)
Worry of patient or family member being misinterpreted of giving up	121(55.8)
Worry of patient or family not accepting	99(45.6)
Unsure of physician’s concerns	76(35.0)
Not sure when is the best timing to discuss	76(35.0)
Lacking related training	68(31.3)
Not familiar to the content of ACP	60(27.6)
Do not know how to communicate with patient or family	55(25.3)
Patients do not feel necessary	39(18.0)

Note: Multiple choices

### Knowledge, attitude, and ACP actions (KAAC-ACP)

The mean score of the 9-item ACP-Knowledge was 4.74 (SD = 1.36), with an accuracy rate of 52.7% (50% as a cut-off point indicated in Zhou’s study). By using the cut-off point of 3 for each item as neutral responses (total score 3*19 = 57 points), the mean score of the three subscales of ACP-Attitude, including ACP belief, subjective standard, and perceived control ranged from 1.81 to 3.72 and the total scores were 48.26 (*p* < 0.001). Similarly, using the cut-off points of 3 for each item as neutral responses (total score 3*4 = 12), the total score of the 3-item ACP-Act was 11.92 (*p* = 0.651). The mean score of the item with ‘I will routinely discuss ACP with end-of-life (EOL) patients’ or ‘I will discuss ACP with over half of EOL patients’ was 3.19 and 3.21 (both *p* < 0.001), respectively, However, there was positive agreement on ‘I will follow up ACP for EOL patients’ (*p* < 0.001).

### Predictors of knowledge, attitude, and ACP actions for nurses

The results from Pearson correlation coefficient among knowledge, attitude, and ACP actions, documented a negative correlation between ACP-K and ACP-A (*p* = 0.002), but no significant correlation between ACP-K and ACP-Action (*p* = 0.69). In contrast, there was significant correlation between overall ACP-A and ACP-Action (*p* < 0.001) and the ACP-A-p (perceived control) had the strongest correlation with ACP-Action (*p* < 0.001) followed by ACP-A-s (subjective standard) (*p* < 0.001) and ACP-A-b (belief) *p* = 0.002). For those significant correlated variables were identified for the simultaneous regression analyses.

According to the significant differences of participants’ characteristics and ACP-K (Tables 4 & 5), there were seven potential predictors, such as age, position, and lacking related training, accounting for variance of R2 15.9% (Adjusted R2 12.3%). Furthermore, results from regression analyses found that the knowledge of ‘head nurses’ was greater than that of ‘nurses’ (*p* = 0.001), the level of knowledge of nurses with ‘7-12 palliative continuous education’ was lower than that with ‘0–6 h’ (*p* = 0.002), and the knowledge level of those who believed they had ‘lacking related training’ was lower than those who did not believe they were lacking relevant training (*p* = 0.014) (Table 6).

**Table 4:** The differences of nurses’ characteristics and knowledge, attitudes, and actions of Advanced Care Planning (ACP) (*n* = 218)

Variables	ACP-Knowledge			ACP-Attitude			ACP-Action		
	M ± SD	*F*	Scheffe’s test	M ± SD	*F*	Scheffe’s test	M ± SD	*F*	Scheffe’s test
Age									
21~30(*n* = 92)	4.47 ± 1.35	3.58*	–	48.94 ± 5.96	0.8		12.12 ± 2.52	1.34	
31~40(*n* = 91)	4.90 ± 1.31			47.85 ± 6.87			11.97 ± 2.88		
41~60(*n* = 35)	5.03 ± 1.15			47.54 ± 6.84			11.26 ± 2.59		
Position title									
RN^a^ (*n* = 201)	4.67 ± 1.31	6.83**	HN > RN	48.44 ± 6.54	1.06		12.03 ± 2.70	3.17*	–
AHN^b^ (*n* = 7)	4.57 ± 0.79		HN > AHN	45.86 ± 7.56			9.57 ± 2.15		
HN^c^ (*n* = 10)	6.20 ± 0.92			46.20 ± 4.37			11.30 ± 1.95		
Clinical ladder									
N^d^(*n* = 77)	4.49 ± 1.37	3.37*	N2 > N	48.87 ± 5.96	1.05		12.14 ± 2.63	0.56	
N1^e^(*n* = 55)	4.65 ± 1.27			48.62 ± 7.05			11.95 ± 3.00		
N2^f^(and)above(*n* = 86)	5.01 ± 1.26			47.48 ± 6.58			11.70 ± 2.54		
Years of work experience									
< 5 years(*n* = 72)	4.46 ± 1.32	4.96*		48.92 ± 5.89	1.11		12.40 ± 2.58	3.55	
> =5 years (*n* = 146)	4.88 ± 1.30			47.93 ± 6.77			11.68 ± 2.72		
Palliative continuous education						0~6 h >			
0~6 h(*n* = 175)	4.87 ± 1.26	4.47*	0~6 h >	48.44 ± 6.22	3.62*	13~21 h	12.02 ± 2.66	0.82	
7–12 h((*n* = 31)	4.19 ± 1.64		7–12 h	49.06 ± 7.54		7–12 h >	11.68 ± 2.44		
13–21 h((*n* = 10)	4.25 ± 0.62			43.50 ± 6.14		13~21 h	11.08 ± 3.66		

a: *RN* registered nurse, b: *AHN* assistant head nurse, c: *HN* head nurse, d: nurses’ working experience within 1 year; e: nurses’ clinical competency at level I defined by Taiwan Nurses Association (can fulfill patients’ basic care); f: nurses’ clinical competency at level II defined by Taiwan Nurses Association (can offer advanced or critical care patients care); ^*^p < 0.05, ^**^p < 0.01

**Table 5:** The influencing factors for nurses’ knowledge, attitude, and actions of Advanced Care Planning (ACP) (*n* = 218)

Variables	ACP-Knowledge			ACP-Attitude			ACP-Action		
	M ± SD	*t*	*p* value	M ± SD	*t*	*p* value	M ± SD	*t*	*p* value
Lacking related training									
yes(*n* = 68)	4.43 ± 1.21	2.382	0.018	49.34 ± 6.74	−1.662	0.098	2.43 ± 2.79	−1.893	0.060
No(*n* = 150)	4.88 ± 1.34			47.77 ± 6.04			11.69 ± 2.62		
Not familiar with the content of ACP									
yes(*n* = 60)	4.50 ± 1.28	1.655	0.099	48.32 ± 6.65	−0.084	0.933	12.80 ± 2.39	−3.043	0.003
No(*n* = 158)	4.83 ± 1.32			48.20 ± 6.46			11.58 ± 2.73		
Patients do not feel necessary									
yes(*n* = 39)	4.90 ± 1.10	− 0.831	0.407	46.90 ± 6.28	2.001	0.047	10.77 ± 2.79	2.997	0.003
No(*n* = 179)	4.70 ± 1.36			48.70 ± 6.48			12.17 ± 2.61		
Unsure of physician’s concerns									
yes(*n* = 76)	4.91 ± 1.27	−1.442	0.151	48.30 ± 6.12	−0.202	0.984	12.47 ± 2.74	−2.190	0.030
No(*n* = 142)	4.64 ± 1.34			48.28 ± 6.00			11.65 ± 2.62		
Not sure when is the best timing to discuss									
yes(*n* = 76)	4.61 ± 1.35	1.094	0.275	49.53 ± 6.43	−2.219	0.034	12.33 ± 2.84	−1.660	0.098
No(*n* = 142)	4.81 ± 1.30			47.58 ± 6.45			11.50 ± 2.59		
Do not know how to communicate with patient or family									
yes(*n* = 55)	4.44 ± 1.30	1.982	0.049	49.09 ± 5.924	1.102	0.272	12.91 ± 2.72	3.232	0.001
No(*n* = 163)	4.84 ± 1.30			7.98 ± 6.67			11.58 ± 2.60		
Worry of patient or family not accepting									
yes(*n* = 99)	4.82 ± 1.34	−0.814	0.416	49.22 ± 6.19	−2.016	0.045	12.56 ± 2.49	−3.267	0.001
No(*n* = 119)	4.67 ± 1.30			47.45 ± 6.66			11.39 ± 2.74		
Worry of patient or family being misinterpreted of giving up									
Yes(*n* = 97)	4.78 ± 1.29	−0.479	0.632	48.57 ± 6.22	−0.795	0.427	12.27 ± 2.49	−2.146	0.033
No(*n* = 121)	4.69 ± 1.36			47.87 ± 6.84			11.49 ± 2.88

**Table 6:** The predicting factors of knowledge, attitude, and action of Advanced Care Planning (ACP) for nurses (*n* = 218)

	unstandardized coefficients		Beta	*t*	*p* value	Collinearity	
	*B*	SE				Tolerance	VIF
ACP-Knowledge							
(constant)	4.807	0.174		27.67	0.000		
Age	0.018	0.037	0.106	0.474	0.636	0.079	12.621
Position title							
RN ^a^(ref ^d^)							
AHN ^b^	−0.296	0.495	− 0.040	−0.598	0.551	0.917	1.091
HN ^c^	1.367	0.421	0.218	3.248	0.001	0.899	1.112
Clinical ladder							
N(ref ^d^)							
N1	0.045	0.235	0.015	0.192	0.848	0.672	1.489
N2(and)above	0.272	0.283	0.101	0.962	0.337	0.366	2.735
Years of work experience	0.009	0.015	0.060	0.644	0.520	0.465	2.152
Palliative continuing education							
0~6 h(ref ^d^)							
7~12 h	−0.774	0.247	−0.206	−3.131	0.002	0.936	1.069
> 12 h	− 0.651	0.375	−0.113	−1.735	0.084	0.954	1.048
lacking related training							
No(ref ^d^)							
Yes	−0.457	0.183	−0.161	−2.490	0.014	0.966	1.035
Do not know how to communicate with patient or family							
No(ref ^d^)							
Yes	−0.211	0.201	−0.070	−1.049	0.295	0.917	1.090
ACP-Attitude							
(constant)	51.966	1.664		31.231	0.000		
ACP-Knowledge	−0.994	0.324	−0.202	−3.072	0.002	0.987	1.013
patients do not feel necessary							
No(ref ^d^)							
Yes	−2.015	1.105	−0.119	−1.823	0.070	0.995	1.005
Not sure when is the best timing to discuss							
No(ref ^d^)							
Yes	1.639	0.891	0.121	1.839	0.067	0.989	1.011
Worry of patient or family not accepting							
No(ref^d^)							
Yes	1.744	0.853	0.134	2.045	0.042	0.990	1.010
ACP-Action							
(constant)	1.097	1.176		0.933	0.352		
ACP-A-b ^e^	0.382	0.062	0.367	6.188	0.000	0.820	1.220
ACP-A-s ^f^	0.161	0.047	0.202	3.452	0.001	0.838	1.193
ACP-A-p ^g^	0.110	0.043	0.146	2.547	0.012	0.876	1.141
Position title							
RN ^A^(ref ^d^)							
AHN ^b^	−1.689	0.840	−0.111	−2.011	0.046	0.945	1.058
HN ^c^	−0.121	0.707	−0.009	−0.171	0.864	0.948	1.055
Not familiar to the content of ACP							
No(ref ^d^)							
Yes	0.581	0.339	0.097	1.715	0.088	0.904	1.107
Patients do not feel necessary							
No(ref ^d^)							
Yes	−0.792	0.390	−0.113	−2.030	0.044	0.926	1.080
Unsure of physician’s concern							
No(ref ^d^)							
Yes	0.748	0.302	0.136	2.478	0.014	0.951	1.052
Do not know how to communicate with patient or family							
No(ref ^d^)							
Yes	0.559	0.348	0.091	1.606	0.110	0.907	1.103
Worry of patient or family not accepting							
No(ref ^d^)							
Yes	0.435	0.306	0.081	1.423	0.156	0.894	1.119
Worry of patient or family being misinterpreted of giving up							
No(ref ^d^)							
Yes	0.344	0.304	0.064	1.130	0.260	0.905	1.105

a: *RN* registered nurse, b: *AHN* assistant head nurse, c: *HN* head nurse, d: *ref* reference group, e: *ACP-A-b* ACP-Attitude-belief, f: *ACP-A-s* ACP-Attitude-subjective standard, g: *ACP-A-p* ACP-Attitude-perceived control

Four potential predictors of ACP-Attitude included ACP-K, ‘patients do not feel necessary’, ‘not sure when is the best timing to discuss’, and ‘Worry of patient or family not accepting’ (Tables 4 & 5) accounted for variance of R2 9.4% (Adjusted R2 7.7%). Of which ACP-K and ‘Worry of patient or family not accepting’ was found to have significant explanatory power, indicating participants with higher ACP-K and lower ACP-A had more positive attitudes (*p* = 0.002) (Table 6). Those who self-reported ‘Worry of patient or family not accepting’ and had higher scores for ACP-A also had more negative attitudes (*p* = 0.042).

In terms of potential predictors for ACP-Action, ten potential predictors found and are shown in Tables 4 & 5 and in the regression analyses, accounted for variance of R2 63.8% (Adjusted R2 37.5%). Six of ten variables had significant variances, such as ACP-A-b (*p* = 0.0001), ACP-A-s (*p* = 0.001), ACP-A-p (*p* = 0.012), position (*p* = 0.046), ‘patients do not feel necessary’ (*p* = 0.044), ‘unsure of physician’s concerns’ (*p* = 0.014) (Table 6). In this model, three subscales of ACP-A become more positive, ACP-Action was more likely to be positive, i.e., ‘Assistant head nurses’ scored lower on AC*P*-Action than ‘nurses’, that is, their ACP-Action was more positive.

## Discussion

This descriptive study is apparently first to explore the implications of ACP, Taiwanese nurses’ knowledge, attitude, actions of ACP, and some of its predictors. The results of this study indicated that 16.1% of physicians actively initiated ACP conversation with patients or their family members. Numerous patients approaching EOL stage would likely rarely or never received hospice care; still, some patients received critical care during the last month of their lives. In addition, nurses’ attitude toward ACP was positive, but their knowledge about ACP and their actions of ACP as measured were suboptimal. In terms of predictors of ACP, the most significant factors included position title and hours of hospice continuing education for knowledge of ACP; level of knowledge and worry of family not accepting for attitude of ACP; and position tile, ACP belief, subjective standard, and perceived control, “unsure of the physician’s concerns about ACP”, and “patients do not feel that ACP actions were necessary” for actions toward ACP.

From a methodological perspective, the selection of a reliable and valid measure is considered to be crucial, especially for a survey study. The measure used to examine nurses’ nurses’ knowledge, attitudes, and actions of ACP in this current study was adapted from a measurement development study by Zhou [21]. The results from Zhou’ s study indicated that a 34-item measure regarding nurses’ knowledge, attitudes and practice behaviors ACP demonstrated fair construct validity and test-retest reliability. Considering the limited studies in the area of ACP for nurses available, thus, the results of this current study would be primarily compared to results from Zhou’ s study.

The majority of nurses in this study reported that physicians occasionally initiated discussions of ACP in their clinical practice, and that the frequency of discussion of ACP with terminal patients was lower than that reported Zhou et al. [21]. In addition, 54% of nurses in the current study believed that terminal patients sometimes received hospice care, but that 63% of such patients died within 2 weeks to 1 month after referral to hospice care, and 65% of nurses believe that terminal patients sometimes (or often) or always received treatment in the intensive care unit within month before death. These results parallel those of Zhou et al. [21], who reported that 62% of their respondents believed that fewer than 50% of patients received hospice care, 47% believed that 50–95% of patients died within 2 weeks of referral to hospice care, and the majority of respondents believed that over 50% of cancer patients underwent chemotherapy sometime within the last month of life. In addition, NHPCO [30] show that 39% of decedents in the United States received hospice care and that 35% of cases died within 7 days of referral. From these data one could make the argument that the timing of referral to hospice care in both the United States and Taiwan is laggard.

The overall ACP-K correct response rate of 53% in this study, indicating that nurses’ knowledge about ACP was prone to be at a moderate level and was lower than that reported by Ryan & Jezewski (68–71% with different measure) [20] and most nurses believed that ADs can effectively communicate patients’ wishes for EOF care. The correct response rate in this study was far lower at 8.3–37.6%, whereas it was 69–81% in Zhou’s study [21]. As mentioned earlier, only having ADs on the medical record without actual conversations with patients cannot ensure that the patient’s wishes are met [31]. This difference might be due to the fact that the Patient Autonomy Act in Taiwan has not yet been implemented [32], so awareness of health care proxies in the ACP implementation process among nurses is not yet well recognized.

The results of ACP-A indicated that nurses’ attitudes toward to ACP were prone to be positive (total scores were 48.26 over 57). Overall, nurses agreed with the importance of ACP (ACP-Ab), but do not fully believe that patients in the EOL require ACP. In addition, nurses were mostly positive about ‘patients want to discuss their own treatment and care’ (ACP-A-s), but were not certain of their responsibility in ACP. This result was similar to that of Izumi [4], who found that over 40% of nurses indicated that they had never participated in ACP and the main reason was that they did not think ACP was their responsibility. Nurses reported significantly more negative attitudes on ACP-A-p in this study and far more negative than a previous study [21]. In particular, nurses in the current study indicated that they were uncomfortable discussing ACP issues with terminal patients, due to their lack of knowledge and confidence. This discrepancy may be because the above mentioned study [21] interviewed APNs, whose clinical role and function differed from those of the nurses in the present study. Another explanation might be related to the dynamics of healthcare team in Taiwan where physicians, more often, are the most informed to make decisions, so nurses feel inappropriate when they want to discuss ACP with patients.

The overall ACP-Act of the nurses in the present study was neutral (total score was 11.92 over 12), but they were less likely to initiate ACP discussion compared to the previous study [21]. However, they were more able to routinely follow up ACP discussions. This might have reflected the nurses’ negative attitudes reported on ACP-A-s (ACP is a professional responsibility for nurses), indicating that nurses may be less likely to engage in routine or active discussion with respect to implementation behaviour, but they are willing to cooperate and participate in the work of tracking ACP. It might be beneficial to use real-life simulation teaching methods in the form of role playing or case studies to enhance the effectiveness of ACP training [33, 34]. In addition, Taiwan is scheduled to implement the Patient Autonomy Law in January 2019 [32] to educate either general publics or healthcare providers to establish ADs and designate medical surrogates early, enhancing the awareness and acceptance of ACP and reducing the resistance by physicians and nurses.

Various predictors of knowledge, attitude, and action for ACP were found in our sample. For example, ACP-Knowledge predictors, ‘position title’ and ‘lacking related training’ of nurses had significant predictive power, and the knowledge of head nurses was significantly greater on the measure use than that of nurses. Because head nurses are more experienced and had policy-driven responsibilities, it is not surprising that they have a more knowledge of ACP compared to line nurses. Factors affecting ACP-A include ACP-K and ‘worry of patient or family not accepting’. As ACP-K increases, ACP-A becomes more positive. This result was consistent with the literature [19], which found that increasing the knowledge of nurses was beneficial to their willingness to promote ACP. Those who reportedly ‘worry of patient or family not accepting’ had more negative ACP-A, potentially crating an obstacle to their implementations of ACP. In addition, the present study found that the ACP-Act of assistant head nurses was significantly greater than that of nurses (*p* = 0.046). Yet, there were no differences between head nurses and nurses (*p* = 0.864). This may be due to the fact that the work of head nurses in Taiwan is more concerned with administrative issues than with frontline patient care, whereas assistant head nurses are usually more experienced than nurses, have more work experience, and are thus more able to implement ACP. This finding was similar to those reported by Black & Emmet [35] and Shepherd [36], which found that clinical experience, personal experience with ADs, and communication skills affected nurses’ implementation of ACP. That investigation also found that older nurses and those with more experience had higher levels of participation in ACP.

## Conclusion

Most of the nurses in the present study believed that it was uncommon for physicians to initiate discussions of ACP in clinical practice, with 65% of respondents indicating that terminal patients continue to receive treatment in intensive care units within 1 month prior to death. In addition, the nurses in our study reported that 63% of patients died within 2 weeks to 1 month after referral to hospice care. Although the nurse respondents in our study reported positive attitudes toward ACP, their knowledge about ACP was suboptimal and the practice of behaviours in ACP discussion was, in our judgment insufficiently proactive. Also, 11 predictors affecting nurses’ knowledge, attitudes, or actions of ACP were identified in this study. In the future, we recommend providing nurses with the on-the-job ACP training program based on those affecting predictors and using situational teaching or case discussion to strengthen nurses’ awareness and communication abilities for ACP professional duties. More importantly, we recommend that nurses practice in accordance with the Taiwanese national policy of promoting the significance of ADs and medical surrogates in the Patient Autonomy Act to improve physicians’ recognition of nurses’ roles in supporting the implementation of ACP, so that the nurses can more confidently play a role in ACP promotion.

### Limitations

Despite some of impressive findings of the nurses’ knowledge, attitudes, and actions toward ACP in this study, any interpretation of this study should consider a number of study limitations. Firstly, the study population is selected on a voluntary basis. The potential nurses not only would potentially introduce selection bias, but also Hawthorne effect is inevitable due to the subjects made a conscious decision to be in the selected hospitals. Secondly, we did not evaluated all the related wards, who might have characteristics that differ from those of the study population, that is, the generalization and external validity should be further discussed. Thirdly, this cross-sectional study obtained measures at a single time point, which might not reflect long-term exposure to the participants’ factors related ACP. Finally, this study was conducted at one regional teaching hospital in southern Taiwan, indicating that no generalizations could be made beyond this hospital in Taiwan and so not for Taiwan itself or wider still globally. Therefore, future investigations with a wider range of regions would make the results more discursive.

## Data Availability

All datasets during and/or analyzed during this study are available from the corresponding author on reasonable request.
